# Vestibular Migraine Following Radiosurgery for Vestibular Schwannoma

**DOI:** 10.7759/cureus.8569

**Published:** 2020-06-11

**Authors:** Ricky Chae, Michael McDermott, Alexander Muacevic, John R. Adler, Jeffrey D Sharon

**Affiliations:** 1 Otolaryngology - Head and Neck Surgery, University of California San Francisco, San Francisco, USA; 2 Neurosurgery, Miami Neuroscience Institute, Miami, USA; 3 Radiosurgery, European CyberKnife Center, Munich, DEU; 4 Department of Neurosurgery, Stanford University School of Medicine, Stanford, USA; 5 Radiation Oncology, Stanford University Medical Center, Stanford, USA

**Keywords:** vestibular migraine, radiosurgery, vestibular schwannoma, venlafaxine

## Abstract

Vestibular schwannoma (VS) is associated with dizziness and vertigo during all stages of treatment. This report describes a patient who presented with a one-year history of intermittent motion sickness, dizziness, headache, imbalance, and nausea. MRI showed a right-side VS in the cerebellopontine angle and internal auditory canal. The patient elected to undergo Gamma Knife radiosurgery for treatment. Within two to three months, she continued to experience recurring dizziness, vertigo, neck stiffness, and head pressure. She was referred for neurotology evaluation, which led to a diagnosis of vestibular migraine (VM). Her vestibular reflexes were intact. Subsequently, she was treated with diet modification and low-dose venlafaxine. She reported dramatically improved dizziness and vertigo symptoms at six-month follow-up. VM is a very common cause of dizziness that should always be included in the differential diagnosis, even in VS patients.

## Introduction

Vestibular schwannoma (VS) is a benign and typically unilateral tumor of the eighth cranial nerve. As such, VS patients commonly present with hearing loss (68%-94%), tinnitus (30%-83%), and vestibular dysfunction (49%-75%), manifesting as dizziness and/or vertigo [1,2]. Using cross-sectional data, Carlson et al. show that over 60% of subjects experience long-term dizziness, with risk factors including female sex, older age, and pre-existing dizziness or migraine [3]. A histologic study has shown that VS is associated with atrophy of the vestibular ganglion, peripheral vestibular nerve branches, and vestibular neuroepithelia [4]. Therefore, it is evident that VS can cause dizziness through direct damage to the vestibular nerves or sensory epithelia.

Vestibular migraine (VM) is increasingly recognized as a common cause of dizziness and vertigo, and diagnostic criteria have been adopted by the Bárány Society and International Headache Society. VM is diagnosed based on having at least five episodes of vestibular symptoms that last 5 minutes to 72 hours, a concurrent or past history of migraine with or without aura, and one or more migrainous features in at least half of the vestibular episodes [5]. VM patients often report worsening of vertigo, imbalance, and disequilibrium with increased physical activity and head movements [6]. In a recent epidemiological study in the United States, 2.7% of the adult population was found to have VM [7].

There are three main treatment modalities for VS: microsurgery, stereotactic radiosurgery (SRS), and observation. Notably, SRS has been suggested as a highly effective and safe option for treating small and medium-sized VS [8]. After surgery or SRS, patients can experience acute vestibular symptoms, including vertigo, dizziness, and imbalance, that significantly impact their quality of life [9,10]. While vestibular hypofunction is the likely cause in many cases, it is always important to maintain a wide differential diagnosis. This report describes a unique VS patient who had recurring dizziness before and after SRS using Gamma Knife. She was subsequently diagnosed with VM, which was successfully managed with diet modification and migraine prophylactic medication.

## Case presentation

A 57-year-old woman initially presented with a one-year history of motion sickness and headache. She also experienced intermittent nausea, dizziness, imbalance, clouded thinking, fatigue, and head pressure. These symptoms often began within one hour of waking up in the morning and could last up to four days. Although she previously attended vestibular physical therapy, she had difficulty performing some of the exercises due to her sensitivity to positional changes. She also had a history of chronic right neck stiffness, headaches with photophobia during periods of nausea and dizziness, and occasional tinnitus and otalgia. She was otherwise healthy, and followed a ketogenic diet and exercise program for weight control. She took the following oral medications: dimenhydrinate 50 mg as needed for motion sickness, zolpidem 10 mg nightly, multivitamin daily, turmeric one capsule daily, magnesium 200 mg daily, and calcium carbonate supplements with vitamin D 625 mg-125 IU twice daily. Her past surgical history includes bunionectomy, coloscopy, and tonsillectomy. She smoked 1/4 pack of cigarettes per day for 2.5 years during her adolescence and would consume 0.8 standard drinks of alcohol per week. There was a family history of Alzheimer’s disease and heart disease. Her review of symptoms was otherwise unremarkable.

**Figure 1:**
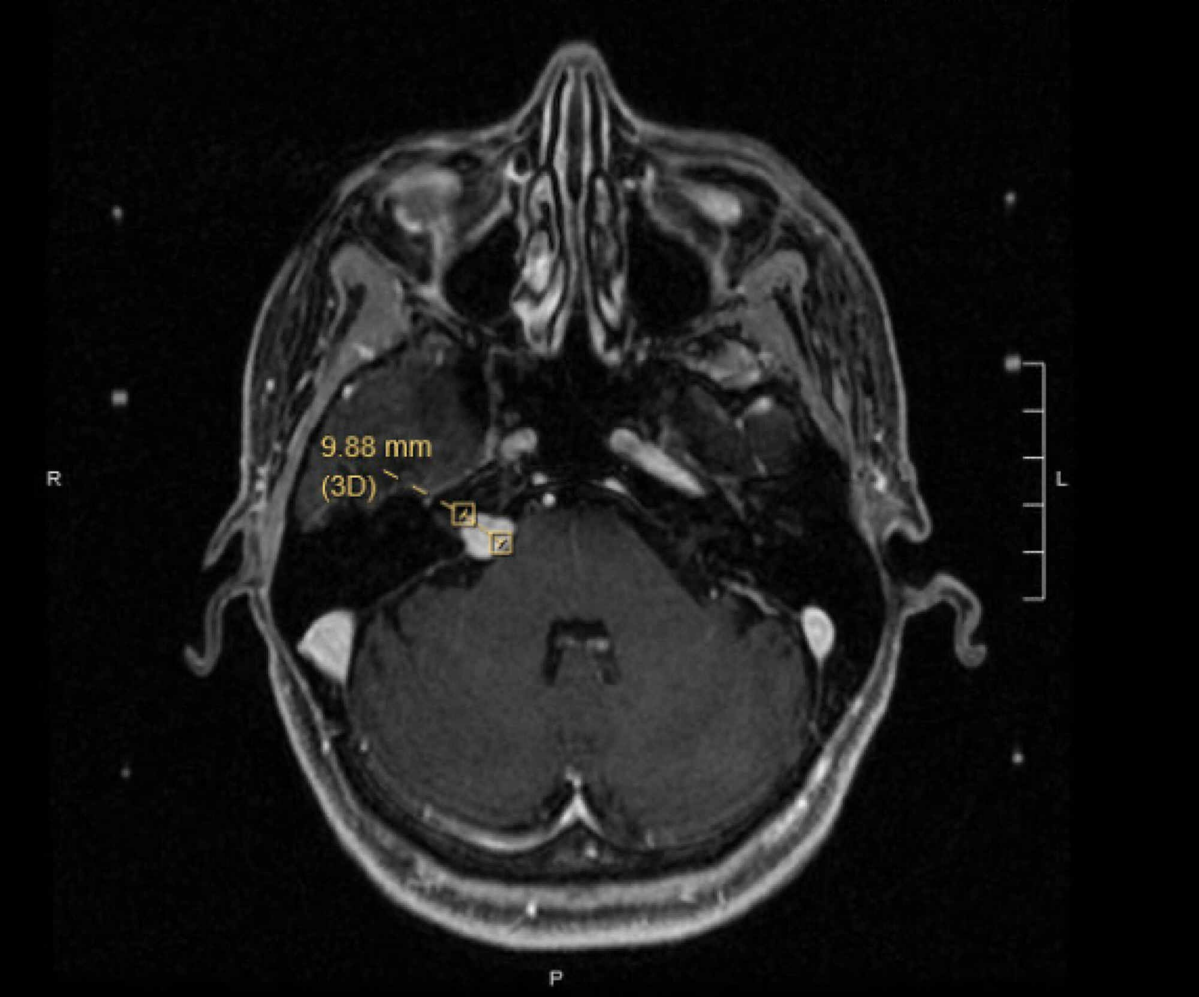
Pre-stereotactic radiosurgery MRI Axial T1 post-contrast image shows a right-side cerebellopontine angle tumor measuring 11 x 12 x 9 mm.

During the initial visit with her primary care provider, the patient reported new nausea with a sudden onset that would occasionally turn into a migraine headache with vertigo. She also reported new mild dyspnea with exertion. As part of her workup, a MRI of her brain was obtained, which showed an 11 x 12 x 9 mm homogeneously enhancing mass at the right cerebellopontine angle and proximal internal auditory canal, suggestive of VS (Figure 1). An audiogram showed normal hearing bilaterally with 100% word recognition score in both ears. She was referred to neurosurgery for evaluation and was prescribed meclizine 25 mg by mouth up to three times daily for dizziness and nausea.

After consideration of the three treatment modalities, she elected to undergo SRS using Gamma Knife. She received 12.5 Gy dose to a target region 11 x 13 x 10 mm encompassing the right VS. She tolerated the treatment well. About a month after SRS, she had recurring dizziness, vertigo, neck stiffness, and head pressure. The dizziness was described as either a spinning sensation or a sense of disequilibrium. She was prescribed diazepam 1 mg by mouth daily for symptomatic relief, but it was not effective. She was also given a course of dexamethasone 2 mg by mouth daily, but that also did not alleviate symptoms. She continued to experience dizziness, vertigo, nausea, intermittent headaches with sensitivity to light and sound, and occasional sharp right ear pain. She reported that these symptoms were affecting her mood as well. 

She was then referred to neurotology for evaluation. Examination of her head and neck did not show any masses. Otomicroscopy was normal. Examination of cranial nerves, including extra-ocular movements, facial movement and sensation, tongue and palate movement, and shoulder shrug, was normal. A 512-Hertz tuning fork was used for the Weber test, which was midline (normal), and the Rinne test, which showed air > bone conduction bilaterally (normal). Vestibular function was assessed with the head impulse test, which was normal. She did not have nystagmus or dizziness in the Dix Hallpike position. Cerebellar examination, including finger to nose testing, rapid alternating movements, and heel to shin testing, was normal. Romberg, with eyes open or closed and with feet together or tandem, showed normal balance. A Timed Up and Go was completed in nine seconds (normal range) with normal gait. Post-treatment audiogram showed no changes compared to baseline. 

With her normal vestibular and cerebellar function, personal history of migraine without aura per International Classification of Headache Disorders, Third Edition (ICHD-3) criteria, and episodic vestibular symptoms associated with headache and light and sound sensitivity, the patient was diagnosed with VM per Bárány Society criteria. Treatment options were discussed, including both pharmacologic and non-pharmacologic paradigms. Due to the severity of symptoms, she elected to do both. A standard handout was provided with recommendations on migraine trigger avoidance, including stress reduction, sleep hygiene, and a migraine elimination diet. The diet change was suggested to avoid any food triggers of her migraines. In addition, medications were discussed with her, and she elected to try venlafaxine 37.5 mg by mouth daily. At first, she reported feeling increased brain fog and head pressure after taking a full tablet of venlafaxine. Therefore, her dose was adjusted to a half tablet.

**Figure 2:**
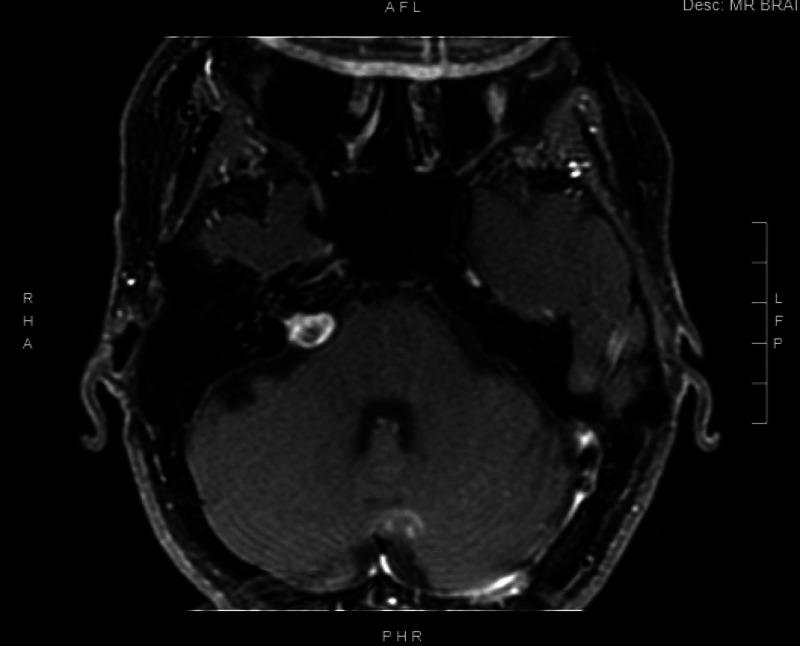
Post-stereotactic radiosurgery (SRS) MRI Axial T1 post-contrast image demonstrating the right vestibular schwannoma with a central necrosis, after SRS using Gamma Knife.

In a follow-up visit three months later, she reported much improved symptoms. Her subjective assessment was that she felt 90% better. She had been avoiding social activities and exercise, which she was able resume after treatment of her VM. Occasionally, certain head movements or positions, such as moving her head up and down, or the “downward dog” yoga position, could trigger symptoms. Her post-SRS MRI showed no significant changes in the size of the right VS, with development of a central necrosis (Figure 2). Further communication six months after treatment showed continued dizziness relief with her treatment regimen.

## Discussion

This report describes a patient treated with SRS using Gamma Knife for a VS, who had recurring dizziness, vertigo, neck stiffness, and head pressure. She was diagnosed and treated for VM, which resulted in significant improvement in her symptoms.

Dizziness in VS patients is a commonly experienced symptom that could be related to a variety of causes other than the tumor itself. In a retrospective cohort study on examining secondary causes of vertigo in VS patients at a tertiary center, Sahyouni et al. found that 78% had comorbid VM [11]. Therefore, it is imperative to evaluate dizziness in VS patients using a wide differential diagnosis as part of their workup and treatment. The nature of symptoms should also be clarified, as dizziness can be used non-specifically. For example, dizziness should be distinguished from pre-syncope, orthostasis, and lightheadedness. Furthermore, many medications can cause dizziness as a side effect. Table 1 summarizes the differential diagnosis for dizziness and vertigo, along with treatment options for each specific diagnosis.

**Table 1:** Differential diagnosis for dizziness in the setting of vestibular schwannoma (VS). The diagnosis, diagnostic clues, and treatment options are listed for distinct vestibular etiologies that should be considered for VS patients experiencing dizziness and vertigo. AICA, anterior inferior cerebellar artery; HIT, head impulse testing; PICA, posterior inferior cerebellar artery; SNRI, serotonin norepinephrine reuptake inhibitor; SSRI, selective serotonin reuptake inhibitor; VEMP, vestibular-evoked myogenic potential; VNG, videonystagmography.

Diagnosis	Diagnostic Clues	Treatment Options
Vestibular migraine	Motion intolerance on exam, light sensitivity	Lifestyle changes, diet modification, migraine prophylactic therapy
Cerebellar dysfunction	Oculomotor dysfunction, dysmetria, ataxia, dysdiadochokinesia	Tumor removal, aminopyridines, vestibular physical therapy
Hydrocephalus	Headache, vomiting, nausea, positional vertigo, MRI showing effacement of fourth ventricle and dilated lateral ventricles	Tumor removal, shunt
AICA stroke	Oculomotor dysfunction, unilateral hearing loss, ataxia, facial weakness, Horner’s syndrome, crossed hemisensory loss	Thrombolysis, anticoagulants, supportive care
PICA stroke	Oculomotor dysfunction, unilateral hearing loss, ataxia, facial weakness, Horner’s syndrome, crossed hemisensory loss, dysphagia	Thrombolysis, anticoagulants, supportive care
Unilateral vestibular hypofunction	Vestibular testing (HIT, VNG, rotary chair testing)	Vestibular physical therapy
Bilateral vestibular hypofunction	Vestibular testing (HIT, VNG, rotary chair testing)	Vestibular physical therapy
Benign paroxysmal positional vertigo	Dix-Hallpike test	Epley maneuver
Superior canal dehiscence syndrome	CT scan, VEMP, audiometry	Surgical plugging or resurfacing of dehiscent canal
Mal de debarquement syndrome	Patient history of disequilibrium (rocking and swaying sensation)	Vestibular physical therapy, stress reduction, SSRI
Ménière’s disease	Audiometry and history	Low salt diet, oral steroids, diuretic, vestibular physical therapy, intratympanic steroids, intratympanic gentamicin
Perilymphatic fistula	Audiometry, VNG, VEMP, history of trauma	Surgical reinforcement of round and oval windows with tissue grafts
Persistent postural perceptual dizziness	Chronic imbalance and dizziness, behavioral assessments	SNRI, SSRI, vestibular physical therapy, cognitive behavioral therapy
Psychiatric causes of dizziness	Behavioral assessments, vestibular testing	Per underlying condition
Vestibular paroxysmia	MRI, audiometry, VEMP, VNG, auditory brain stem test	Oxcarbazepine, carbamazepine
Presbyvestibulopathy	HIT, postural instability, recurrent falls, vestibular testing, gait disturbance	Vestibular physical therapy

First, dangerous causes of dizziness must be ruled out. These include hydrocephalus and cerebellar stroke, which can involve an acute onset of dizziness. VS patients with associated hydrocephalus may present with headache, vomiting, nausea, positional vertigo, and MRI showing effacement of the fourth ventricle and dilated lateral ventricles. Treatment options include ventricular shunting and tumor removal. With anterior inferior cerebellar artery (AICA) stroke, unilateral hearing loss, vestibular dysfunction, gait and limb ataxia, dysmetria, and facial weakness are possible. Since branches of AICA supply the eighth cranial nerve, auditory and vestibular dysfunction is widely reported [12]. With posterior inferior cerebellar artery (PICA) stroke, vestibular dysfunction is usually observed, together with nausea, vomiting, vertigo, dysmetria, and dysarthria. In both AICA and PICA stroke, direction-changing nystagmus and severe ataxia are important clinical clues.

Cerebellar dysfunction, which can be caused by mass effect from the tumor, manifests as dizziness and vertigo in 80% of chronic cases and 40%-60% in acute cases [13]. Physical exam findings can include gaze-evoked nystagmus, saccadic dysmetria, and impaired vestibulo-ocular reflex cancellation. In addition, patients with cerebellar dizziness and vertigo frequently present with coordination problems, such as dysmetria and dysdiadochokinesia. Treatment of cerebellar dysfunction associated with VS involves expeditious removal of the tumor. Post-treatment cerebellar dysfunction involves consideration of physical therapy and pharmacologic treatments, including aminopyridines, chlorzoxazone, and N-acetyl-DL-leucine.

With vestibular hypofunction, asymmetries in vestibular afferents result in vertigo and imbalance. This condition can be subdivided into static and fluctuating vestibular dysfunction. With static dysfunction, vestibular physical therapy has been shown to be instrumental in recovering loss of function [14]. Importantly, patients undergoing vestibular rehabilitation should avoid vestibular suppressants and be physically active. For patients who are not progressing with vestibular physical therapy, providers should assess vision, including stereoscopy, lower extremity sensation, and contralateral vestibular function. The latter is particularly important to consider even before treatment intervention, as contralateral loss may have implications for treatment decision making. This can be assessed at bedside using the clinical head impulse test. With fluctuating vestibular dysfunction resistant to the aforementioned treatments, it is worth considering ablative treatments, such as intratympanic gentamicin for unilateral deafferentation of the vestibular system. While this procedure can be done after SRS and for tumors being observed, formal vestibular testing should always be performed in advance.

Additionally, vestibular hypofunction can occur unilaterally or bilaterally. Unilateral hypofunction may arise from comorbid vestibular neuritis, VS, treatment for VS, Ménière’s disease, and treatment for Ménière’s disease. Bilateral hypofunction may arise from damage to the eighth cranial nerve due to neurofibromatosis type II, autoimmune diseases, trauma, congenital problems, aminoglycoside antibiotics, idiopathic causes, superficial siderosis, and CANVAS syndrome (cerebellar ataxia, neuropathy, vestibular areflexia). Although both types of vestibular hypofunction involve the same diagnostic clues and treatment, patients with bilateral hypofunction will often experience longer and more difficult vestibular physical therapy.

Patients should also be evaluated for other common vestibular pathologies [15]. Benign paroxysmal positional vertigo (BPPV) involves episodes of dizziness and spinning sensation that last under a minute. The most common variant is posterior canal BPPV, which is treated with the Epley maneuver. VM is another prevalent yet widely underdiagnosed vestibular pathology [7]. Since there are no disease markers on exam or ancillary testing for VM, the Bárány Society criteria are widely used to diagnose VM. Finally, rare causes of dizziness include superior canal dehiscence syndrome, mal de debarquement syndrome, Ménière’s disease, perilymphatic fistula, persistent postural perceptual dizziness, psychiatric causes of dizziness, vestibular paroxysmia, and presbyvestibulopathy. Superior canal dehiscence syndrome can be definitively diagnosed through a combination of CT scan, vestibular-evoked myogenic potentials, audiometry testing, and characteristic symptoms, including pulsatile tinnitus, autophony, and sound and/or pressure-induced dizziness. Treatment involves surgical plugging or resurfacing of the dehiscent canal.

Dizziness is one of the most significant predictors of quality of life in VS patients [10,16,17]. Lee et al. report that 32% of VS patients developed vestibular dysfunction within six months of SRS [18]. This highlights the importance of dizziness evaluation and treatment as part of the comprehensive management of VS. A recent interventional study reports that all patients with VS and comorbid VM had complete symptomatic resolution of their vertigo after being treated with migraine prophylactic therapy (e.g., verapamil, topiramate, nortriptyline) and undergoing migraine diet and lifestyle changes [11]. In a non-placebo randomized controlled trial, both venlafaxine and propranolol were found to reduce vertigo episodes and severity in VM patients. Venlafaxine additionally led to decreased depressive symptoms [6,19]. In a larger study of subjects with migraine-associated dizziness, nortriptyline combined with dietary manipulation (avoidance of processed cheese, meat, and red wines) was found to completely resolve or reduce symptoms in 77% of subjects undergoing this treatment regimen [20]. This improvement from a combined treatment of diet changes and migraine prophylactic medication is consistent with the findings in our report.

## Conclusions

This report highlights the significance of carefully evaluating dizziness using a wide differential diagnosis in the setting of VS. Our patient was diagnosed with VM, and subsequent diet modification along with pharmacologic treatment led to a significant reduction in symptoms.
